# FBXO43 increases CCND1 stability to promote hepatocellular carcinoma cell proliferation and migration

**DOI:** 10.3389/fonc.2023.1138348

**Published:** 2023-03-03

**Authors:** Chun-Ming Li, Jie Zhang, Wu Wu, Zhu Zhu, Feng Li, Di Wu, Xiao-Jun Wang, Chuan-Ming Xie, Jian-Ping Gong

**Affiliations:** ^1^ Department of Hepatobiliary Surgery, The Second Affiliated Hospital of Chongqing Medical University, Chongqing, China; ^2^ Key Laboratory of Hepatobiliary and Pancreatic Surgery, Institute of Hepatobiliary Surgery, Southwest Hospital, Third Military Medical University (Army Medical University), Chongqing, China; ^3^ Department of Hepatobiliary Surgery, The Third Affiliated Hospital of Chongqing Medical University, Chongqing, China

**Keywords:** FBXO43, CCND1, hepatocellular carcinoma (HCC), proliferation, migration

## Abstract

**Background and Aims:**

Abnormal expression of E3 ubiquitin ligase plays an important role in the development and progression of hepatocellular carcinoma (HCC), although the mechanism has remained elusive. This study aimed to investigate the biological function and potential mechanism of FBXO43 in HCC.

**Methods:**

FBXO43 expression in tissues and cells were detected by quantitative real-time PCR (qRT−PCR), Western blot, and immunohistochemistry (IHC). The Kaplan-Meier method and Cox regression analysis were used to explore the correlation between the expression level of FBXO43 and the clinical survival. MTT assay, EdU incorporation, colony formation, Transwell, and wound healing assays were performed to evaluate the function of FBXO43 in cell proliferation and migration *in vitro*. The interaction between FBXO43 and cyclin D1 (CCND1) was assessed by co-immunoprecipitation (Co-IP) assay and *in vivo* ubiquitination assay.

**Results:**

We found that FBXO43 was upregulated in HCC patient tissues and positively associated with poor clinicopathological features. Meanwhile, HCC patients with high expression of FBXO43 had shorter overall survival (OS) and disease-free survival (DFS). Furthermore, knockdown of FBXO43 inhibited HCC cell proliferation, migration and epithelial-mesenchymal transition (EMT) in HCC cells. Mechanistically, FBXO43 interacted with CCND1 and promoted its stability by polyubiquitination, leading to HCC cell proliferation, migration and EMT. Functional rescue experiments demonstrated that knockdown of CCND1 blocks FBXO43-mediated cell proliferation and metastasis.

**Conclusions:**

FBXO43, as an independent prognostic biomarker, promotes HCC cell proliferation, metastasis and EMT by stability of CCND1, which provides a new potential strategy for HCC treatment by targeting FBXO43-CCND1 axis.

## 1 Introduction

Hepatocellular carcinoma (HCC) is the sixth most common cancer worldwide and the third leading cause of cancer-related death (1). However, the specific mechanisms underlying HCC development and progression remain unclear. Surgical resection is still the main treatment for liver cancer but once patients have symptoms and signs, the disease has progressed to the late stages and patients lose the chance of radical surgery. However, current imaging examinations and alpha-fetoprotein (AFP) detection lack sensitivity and specificity for early diagnosis. Therefore, it is urgent to find novel biomarkers for the diagnosis and targets for treatment of HCC.

FBXO43, also named EMI2, ERP1, FBX43, OOMD12, and SPGF64, is located on 8q22 and is a member of the F-box protein family. F-box protein, as a component of E3 ubiquitin ligase in the ubiquitin-proteasome system (UPS), is responsible for the selective recognition of substrate proteins during ubiquitination (2). Some members of the F-box protein family, including FBXO45 (3), FBXW10 (4), and β-TRCP (5) have been reported to be involved in occurrence and progression of HCC. FBXO43 is involved in establishing and maintaining the arrest of oocytes at the second meiotic metaphase until fertilization by inhibiting the anaphase-promoting complex/cyclosome (APC/C) ubiquitin ligase (6). A recent study reported that FBXO43 played a key role in promoting breast cancer growth (7). A study based on bioinformatics analysis revealed that FBXO43 was overexpressed in HCC and associated with poor survival (8). However, whether FBXO43 may be a potential independent prognostic factor and the role of FBXO43 in HCC development remains unclear.

Cyclins are a protein family that regulate cell cycle progression. Cyclin D1 (CCND1), a subtype of cyclin D, forms complexes with cyclin-dependent kinase 4/6 (CDK4/6) and triggers phosphorylation of the retinoblastoma protein (Rb) to release the transcription factor E2F and promote the G1-to-S phase transition (9, 10). It has a very short half-time because of the PEST domain for rapid degradation to insure the normal progression of the cell cycle and cell proliferation (11). However, CCND1 is often abnormal expressed in many cancers, including HCC. CCND1-dependent activation of Smad2/3 modulates liver cancer stem cells self-renewal and controls HCC progression (12). Moreover, recent studies have shown that CCND1 induced migration and epithelial-mesenchymal transition (EMT) in breast cancer (13). CCND1 ubiquitination by ROC1-CUL1 and FBX4 has been reported to be involved in the progression of cancers (14) but how the CCND1 protein is modulated in HCC is largely unknown.

In this study, we found that FBXO43 was upregulated in HCC patients and HCC cell lines. High expression of FBXO43 is an independent risk factor for poor prognosis in HCC patients. Knockdown of FBXO43 inhibited the proliferation, migration, and EMT of HCC cell lines. Mechanistically, FBXO43 interacted with CCND1 and promoted its stability by polyubiquitination, leading to HCC cell proliferation, migration and EMT. Therefore, our study provides a significant biomarker for the diagnosis and target for the treatment of HCC.

## 2 Materials and methods

### 2.1 Patients and tissue samples

Tumor tissues (T) and adjacent normal tissues (N) were collected from HCC patients who were hospitalized in the Department of Hepatobiliary Surgery, Southwest Hospital, Third Military Medical University, for subsequent mRNA and protein detection. Written informed consent for the collection of clinical samples was obtained from the patients and their families. The study was approved by the ethics committee of the Southwest Hospital, Third Military Medical University and complied with the Declaration of Helsinki.

### 2.2 Cell culture and transfection

The five HCC cell lines (LM3, HepG2, Hep3B, SMMC-7721, and Huh7) and the normal hepatocyte cell line LO2 were derived from the Institute of Biochemistry and Cell Biology (Shanghai, China). All cell lines were cultured with Dulbecco’s modified Eagle’s medium (Gibco, USA) containing 10% fetal bovine serum (Gibco, USA) and 100 units/ml penicillin and streptomycin (HyClone, USA) in a humidified incubator with 5% CO2 at 37°C. Lipofectamine 2000 (Invitrogen) was used for FBXO43 small interfering RNA (siRNA), CCND1 siRNA or negative control siRNA transfection based on the manufacturer’s instructions. FBXO43 siRNA-1 (5’-CAAGTTATCAACTTAGAAA-3’), FBXO43 siRNA-2 (5’-TTAACACATCCTTTAGAAT-3’), CCND1 siRNA (5’- CCACAGATGTGAAGTTCATTT-3’), and its associated control siRNA were obtained from GenePharma (Shanghai, China).

### 2.3 Quantitative real-time PCR

Total RNA was extracted from HCC patient tissues and HCC cell lines using Trizol Reagent (Invitrogen, 15596018) according to a previously described protocol (15). qRT−PCR was used to measure the tissues and cells mRNA expression levels using TB Green Premix Ex Taq II (TaKaRa). β-actin was used as the internal control. The experimental sequence primers used are as follows: 5’-CTCCGATAAGTAATCTTGTGGC-3’ (forward) and 5’-CTTGTCTTTCTTATGGTGTCCC-3’ (reverse) for FBXO43, 5’-TCAGGCGTCTGTAGAGGCTT-3’ (forward) and 5’-ATGCACATCCTTCGATAAGACTG-3’ (reverse) for N-Cadherin, 5’-CGAGAGCTACACGTTCACGG-3’ (forward) and 5’-GGGTGTCGAGGGAAAAATAGG-3’ (reverse) for E-Cadherin, 5’-AGTCCACTGAGTACCGGAGAC-3’ (forward) and 5’-CATTTCACGCATCTGGCGTTC-3’ (reverse) for Vimentin, and 5’- CCTTCCTGGGCATGGAGTCCT-3’ (forward) and 5’- GGAGCAATGATCTTGATCTT -3’ (reverse) for β-actin.

### 2.4 Western blot assay

The total proteins of HCC tissues or cells were extracted using RIPA lysis buffer (Beyotime, China). The samples were incubated on ice for 15 mins, and cell debris was removed after 12,000 g at 4°C for 10 mins. The protein concentration was determined using a bicinchoninic acid kit (Beyotime, China) according to the manufacturer’s instructions. After adding 5 × loading buffer, the proteins were separated by polyacrylamide gel electrophoresis (PAGE) and transferred to a nitrocellulose transfer membrane (GE Healthcare, USA). The membranes were incubated overnight at 4°C with the following specific primary antibodies: FBXO43 (Proteintech, 55176-1-AP, 1:1000), β-actin (Proteintech, 60008-1-Ig, 1:5000), Ki-67 (CST, 9449, 1:1000), Vimentin (CST, 3932, 1:1000), N-Cadherin (CST, 14215, 1:1000), E-Cadherin (CST, 14472, 1:1000), and CCND1 (CST, 2978, 1:1000). After washing three times with TBST, the membranes were incubated with anti-rabbit immunoglobulin G (IgG) (CST, 7074, 1:5000) or anti-mouse immunoglobulin G (IgG) (CST, 7076, 1:5000) secondary antibody at room temperature for 1hrs. After washing again, the protein bands were visualized using an Image Analysis System (Bio-Rad, USA) with an Enhanced Chemiluminescence Fluorescence Detection kit (GE HealthCare, USA).

### 2.5 Immunohistochemistry

Paraffin-embedded tissues from HCC patients were cut into three µm slices. Antigen repair was performed by high temperature and high pressure with sodium citrate solution (Beyotime, China). Endogenous peroxidase was inactivated with 3% hydrogen peroxide solution for 10 mins and then blocked with goat serum at room temperature for 30 mins. Then, the sections were incubated overnight at 4°C with the following specific primary antibodies: Ki-67 (CST, 9449, 1:800), FBXO43 (Proteintech, 55176-1-AP, 1:400), and CCND1 (CST, 2978, 1:100). After incubating goat anti-mouse or goat anti-rabbit secondary antibodies at room temperature for 30 mins, immunohistochemistry (IHC) was performed using a polymer horseradish peroxidase detection system (Zhongshan Goldenbridge Biotechnology, China). The positive staining was observed and photographed by an inverted microscope, and positive cells of the staining were counted by ImageJ.

### 2.6 MTT assay

The MTT assay was used to analyze cell proliferation. Huh7 and LM3 cells were seeded at 3000 cells/well in 96-well plates. 10ul of MTT reagent (5mg/mL, Solarbio, China) was added to each well at three time points (24, 48, and 72 hrs) after transfection and incubated at 37 °C for 4 hrs. Then, the culture medium was removed from the wells, and 100 µl DMSO was added to each well to dissolve the formazan. The absorbance at 490 nm was detected to assess cell proliferation by using an enzyme calibration system (ThermoFisher Scientific, USA).

### 2.7 5-Ethynyl-2’-deoxyuridine incorporation assay

The 5-ethynyl-2’-deoxyuridine (EdU) (Beyotime, China) assay was used to assess cell proliferation according to the manufacturer’s instructions. Briefly, Huh7 and LM3 (5 × 10^4^ cells/well) cells were cultured in 24-well plates for 24 hrs. Then, the cells were incubated with preheated 10ul EdU at 37°C for 2 hrs. The cells were treated with 4% formaldehyde for 15 mins and were permeated with 0.3% Triton X-100 solution for 10 mins. The click additive solution was used to stain the cells for 30 mins and 1× Hoechst 33342 staining was added to stain the nuclei for 10 mins in the dark. The rate of EdU-positive cells was calculated by ImageJ.

### 2.8 Colony-forming assay

Huh7 and LM3 cells (500 cells/well) were seeded in 6-well plates and cultured for 2-3 weeks to form a colony composed of at least 50 cells. The colonies were fixed in 4% formaldehyde for 15 mins. After washing with PBS, the colonies were stained with 0.1% crystal violet for 30 mins and the colony numbers were counted by microscopy.

### 2.9 Wound healing assay

Huh7 and LM3 cells (5 × 10^5^ cells/well) were grown in 6-well plates until complete confluence. Then, a 200 µL pipette tip was used to scratch the cells, and PBS was used to remove damaged cells. After 0, 24, and 48 hrs, the scratch was photographed using an inverted microscope, and the wound area was measured by ImageJ.

### 2.10 Transwell assay

The 24-well Transwell chambers (Corning, USA) were used to perform Transwell assays. The lower chamber was filled with 500 µL DMEM containing 10% FBS, while the upper chamber was filled with 200 µL DMEM containing 1× 10^5^ cells. After 24 hrs, the Transwell chambers were fixed in 4% formaldehyde for 15 mins and stained with 0.1% crystal violet for 10 mins. The migrated cells were observed and photographed by inverted microscopy, and the number of migrating cells was measured by ImageJ.

### 2.11 Co-immunoprecipitation assay

Huh7 and LM3 cells were transfected with 3 × Flag-FBXO43 for 48h. Then, the cells were lysed in cell lysis buffer for Western and IP (Beyotime, China) on ice for 20 mins, and cell dewas were removed after 12,000 g at 4°C for 10 mins. The 5ug primary antibodies and 40ul Protein A/G PLUS-Agarose (Santa Cruz, CA) were added to the cell lysates and rotating incubated at 4°C overnight. The beads were washed four times with IP lysis buffer (20 mM Tris-HCl, pH 8.0,100 mM NaCl, and 1% NP-40), and the 2× loading buffer (Beyotime, China) was added to the bead and were boiled for 10 mins. The supernatant was used in Western blot assay.

### 2.12 Statistical analysis

GraphPad Prism Version 8.0 (CA, USA) and SPSS 25 Software (SPSS Inc., USA) were used to analyze the trial data. The Pearson χ2 test was used to evaluate the relationship between the FBXO43 expression level and clinicopathological features. Linear regression was used to analyze the association between the two continuous variables. Cox regression analysis was used for univariate and multivariate analyses. The Kaplan-Meier method was performed for survival analysis. The t-test was used to compare the differences between the two groups, and analysis of variance (ANOVA) was used when more than two groups were compared. All measurement data were expressed as the mean ± SEM. p< 0.05 indicated statistical significance.

## 3 Results

### 3.1 FBXO43 is highly expressed in HCC patients and positively correlates with poor survival

**Figure 1:**
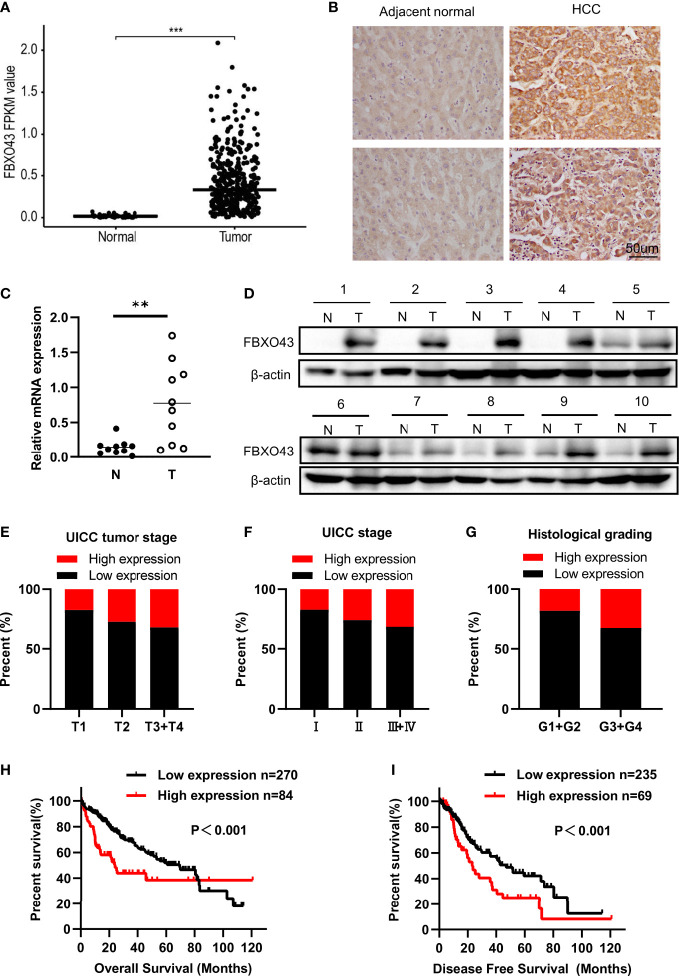
FBXO43 is highly expressed in HCC patients and positively correlated with poor survival. **(A)** The expression levels of FBXO43 in HCC (n=374) and adjacent normal tissues (n=50) in the TCGA database. **(B)** Immunohistochemical staining revealed that FBXO43 was upregulated in HCC tissues (n=20). **(C)** Quantitative real-time PCR (qRT−PCR) and **(D)** Western blot were used to measure the mRNA and protein expression of FBXO43 in 10 pairs of HCC tumor tissues (T) and adjacent normal tissues (N). **(E–G)** The association between FBXO43 and UICC tumor stage **(E)**, UICC stage **(F)**, or histological grading **(G)** based on HCC TCGA database. **(H, I)** Kaplan-Meier curves showing the correlation between FBXO43 and overall survival (OS) **(H)** or disease-free survival (DFS) **(I)**. Data were from the TCGA database. Two-tailed t-test was used. **p<0.01, ***p<0.001.

Consistent with a study of gene co-expression network analysis in HCC (8), we found that the mRNA expression level of FBXO43 was significantly increased in 374 HCC tumor tissues compared with 50 normal tissues in the TCGA database (**Figure 1A**). IHC was used to detect the protein expression of FBXO43 in 20 HCC tissues and paired adjacent tissues, and we found that FBXO43 was upregulated in 75% HCC (**Figure 1B**). Meanwhile, both mRNA and protein levels of FBXO43 were increased in 10 HCC tissues compared with paired adjacent tissues (**Figures 1C, D**). In addition, the correlation between FBXO43 expression and clinicopathological characteristics was analyzed. We found that the FBXO43 expression level was positively associated with poor clinicopathological features, including UICC tumor stage (Pearson χ2 test, p=0.018), UICC stage (Pearson χ2 test, p=0.039), histological grade (Pearson χ2 test, p=0.010), vascular invasion (Pearson χ2 test, p=0.000), and adjacent inflammation (Pearson χ2 test, p=0.039) (**Figures 1E–G**; **Table 1**).

**Table 1:** Correlation between the clinicopathologic variables and FBXO43 expression in HCC patients.

Variable	All cases	Low expression		High expression		P-value*
		n	%	n	%	
Age(years)						
<60	161	117	72.7	44	27.3	0.146
≥60	193	153	79.3	40	20.7	
Gender						
Male	239	182	76.2	57	23.8	0.939
Female	115	88	76.5	27	23.5	
UICC tumor stage						
T1	174	143	82.2	31	17.8	**0.018**
T2+T3+T4	177	124	70.1	53	29.9	
Tx	3	3	100.0	0	0.0	
Lymph node metastasis						
Negative	240	184	76.7	56	23.3	0.907
Positive	3	2	66.7	1	33.3	
Nx	111	84	75.7	27	24.3	
Distant metastasis						
Negative	255	194	76.1	61	23.9	0.625
Positive	3	3	100.0	0	0.0	
Mx	96	73	76.0	23	24.0	
UICC stage						
I	165	136	82.4	29	17.6	**0.039**
II+III+IV	165	117	70.9	48	29.1	
Data unavailable	24	17	70.8	7	29.2	
Histological grading						
G1 +G2	221	180	81.4	41	18.6	**0.010**
G3 + G4	128	86	67.2	42	32.8	
Gx	5	4	80.0	1	20.0	
Vascular invasion						
Negative	198	160	80.8	38	19.2	**0.000**
Positive	102	80	78.4	22	21.6	
Data unavailable	54	30	55.6	24	44.4	
Resection status						
R0	312	238	76.3	74	23.7	0.741
R1 +R2	17	14	82.4	3	17.6	
Rx	25	18	72.0	7	28.0	
Family history						
Negative	198	149	75.3	49	24.7	0.322
Positive	109	88	80.7	21	19.3	
Data unavailable	47	33	70.2	14	29.8	
Adjacent inflammation						
None	114	92	80.7	22	19.3	**0.039**
Mild	95	74	77.9	21	22.1	
Severe	17	16	94.1	1	5.9	
Data unavailable	128	88	68.8	40	31.3	

*χ^2^-test, P < 0.05 was considered for statistic significance, and was marked in bold. HCC, hepatocellular carcinoma; UICC, Union for International Cancer Control.

Then, we explored the correlation between the expression level of FBXO43 and the clinical survival. Patients with high expression of FBXO43 had shorter OS (p<0.001) and DFS (p<0.001) (**Figures 1H, I**). Cox regression analysis was used to further explore the relationship between FBXO43 and the OS of HCC patients. After univariate Cox regression, FBXO43 mRNA expression levels, UICC tumor stage, distant metastasis, UICC stage, vascular invasion, resection status, and adjacent inflammation were defined as potential risk factors for OS (**Table 2**). Multivariate Cox regression analysis showed that FBXO43 mRNA expression levels, UICC tumor stage, resection status, and adjacent inflammation were independent risk factors for OS of HCC patients (**Table 3**). Therefore, high expression of FBXO43 is an independent risk factor for poor survival in HCC patients.

**Table 2:** Univariate analysis indicating associations between overall survival and various risk factors.

Variable	All cases	Mean	Median	P-value
Age(years)				
<60	161	67.068	69.510	0.292
≥60	193	56.075	48.950	
Gender				
Male	239	67.030	81.670	0.277
Female	115	54.410	48.950	
UICC tumor stage				
T1	174	73.404	80.680	**0.000**
T2+T3+T4	177	49.250	37.290	
Tx	3	37.680	37.680	
Lymph node metastasis				
Negative	240	70.394	83.510	0.053
Positive	3	25.330	33.020	
Nx	111	41.132	37.680	
Distant metastasis				
Negative	255	68.717	80.680	**0.004**
Positive	3	18.747	18.330	
Mx	96	41.509	37.290	
UICC stage				
I	165	75.861	83.180	**0.000**
II+III+IV	165	51.740	39.750	
Data unavailable	24	33.389	27.500	
Histological grading				
G1 +G2	221	60.976	58.840	0.423
G3 + G4	128	66.086	53.290	
Gx	5	37.490	37.680	
Vascular invasion				
Negative	198	68.396	80.680	**0.000**
Positive	102	57.700	48.950	
Data unavailable	54	34.805	21.320	
Resection status				
R0	312	64.675	60.840	**0.000**
R1 + R2	17	40.587	37.290	
Rx	25	16.147	19.090	
Family history				
Negative	198	63.981	83.180	0.507
Positive	109	54.998	45.070	
Data unavailable	47	62.448	60.840	
Adjacent inflammation				
None	114	73.368	80.680	**0.000**
Mild	95	55.364	NR	
Severe	17	62.359	NR	
Data unavailable	128	36.627	27.500	
FBXO43 (FPKM value)				
<0.56	270	63.147	69.510	**0.000**
≥ 0.56	84	55.086	23.780	

NR, not reached. P < 0.05 was considered for statistic significance, and was marked in bold.

**Table 3:** Multivariate analysis indicating associations between overall survival and various risk factors.

Variable	β	SE	Hazard ratio	95%CI	P-value
UICC tumor stage	0.573	0.292	1.774	1.002-3.143	**0.049**
Distant metastasis	0.196	0.110	1.217	0.981-1.509	0.074
UICC stage	0.053	0.232	1.054	0.669-1.663	0.820
Vascular invasion	0.112	0.125	1.119	0.875-1.429	0.370
Resection status	0.36	0.170	1.435	1.027-2.004	**0.034**
Adjacent inflammation	0.314	0.077	1.369	1.178-1.591	**0.000**
FBXO43 (FPKM value)	0.663	0.200	1.941	1.312-2.872	**0.001**

β, Regression coefficient; SE, standard error; CI, confidence interval. P < 0.05 was considered for statistic significance, and was marked in bold.

### 3.2 Decreased expression of FBXO43 can inhibit the proliferation of HCC cells

**Figure 2:**
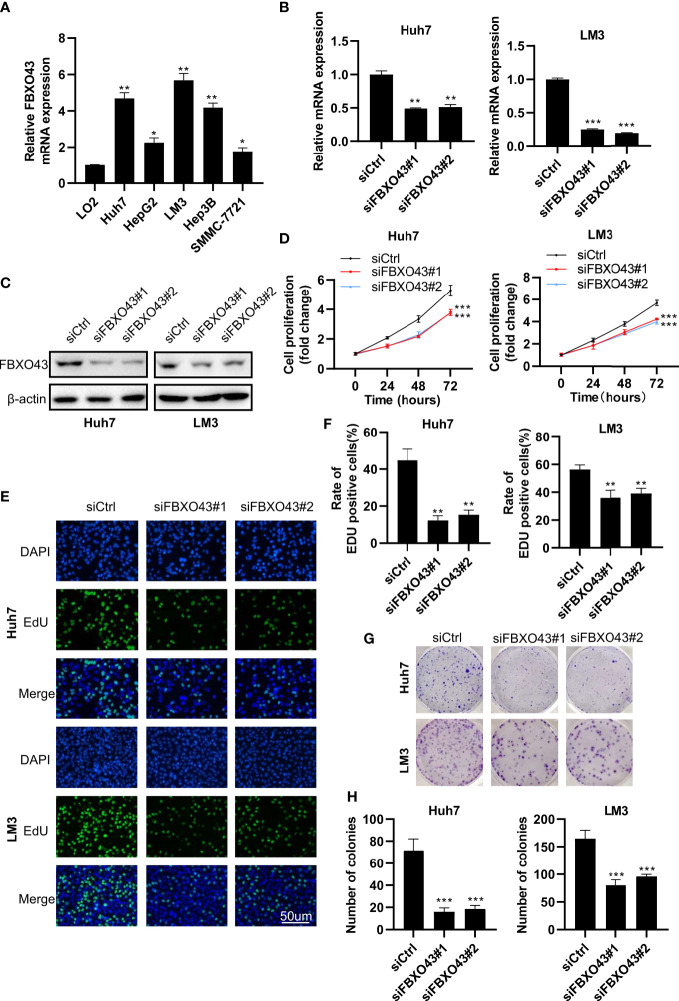
Knockdown of FBXO43 inhibits HCC cell proliferation in HCC cell lines. **(A)** Relative mRNA expression of FBXO43 in 5 HCC cell lines (Huh7, HepG2, LM3, Hep3B, and SMMC-7721) compared with the normal hepatocyte cell line LO2. **(B–H)** Huh7 and LM3 cells were transfected with siControl (siCtrl) or siRNA targeting FBXO43 (siFBXO43) for 48hrs. qRT−PCR **(B)** and Western blot **(C)** were used to measure the mRNA and protein expression of FBXO43. **(D–H)** Cell proliferation was analyzed in Huh7 and LM3 cells by MTT assay **(D)**, 5-ethynyl-2’-deoxyuridine (EdU) incorporation assay **(E, F),** and colony formation assay **(G, H)**. Data were expressed as Mean ± SEM. One-way ANOVA with Tukey’s multiple comparisons test was used. *p<0.05, **p<0.01, ***p<0.001.

**Figure 3:**
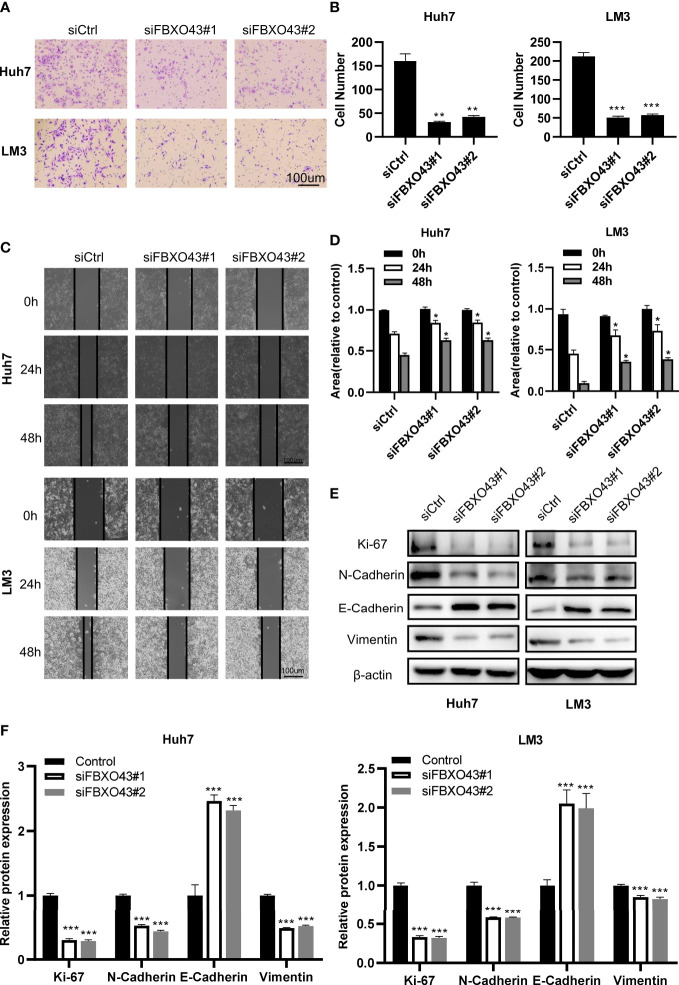
Knockdown of FBXO43 inhibits the migration and epithelial-mesenchymal transition (EMT) of HCC cell lines. **(A–D)** Huh7 and LM3 cells were transfected with siCtrl or siFBXO43 for 48hrs. Cell migration was analyzed by Transwell assay **(A, B)** and wound healing assay **(C, D)**. **(E, F)** Huh7 and LM3 cells were transfected with siCtrl or siFBXO43 for 48 hrs and then lysed. The protein expression of proliferation- and EMT-associated markers was measured by Western blot. Data were expressed as Mean ± SEM. One-way ANOVA with Tukey’s multiple comparisons test was used in **(B)** Two-way ANOVA with Bonferroni’s multiple comparisons test was used in D and **(F)** *p<0.05, **p<0.01, ***p<0.001.

Since the expression level of FBXO43 is closely related to UICC stage and vascular invasion, we speculate that FBXO43 may be involved in the proliferation and metastasis of HCC. To explore the role of FBXO43 in the proliferation of HCC, we first detected the expression levels of FBXO43 in five HCC cell lines and a normal liver cell line by qPCR. Compared with the normal hepatocyte cell line LO2, the expression levels of FBXO43 were increased at different levels in the five HCC cell lines (**Figure 2A**). Huh7 and LM3 cells with high expression levels of FBXO43 were used to study the role of FBXO43 in cell proliferation in HCCs by knockdown of FBXO43. The results revealed that knockdown of FBXO43 significantly reduced FBXO43 expression and inhibited cell proliferation, as indicated by MTT assay (**Figures 2B–D**). Furthermore, knockdown of FBXO43 significantly reduced EdU incorporation and colony formation (**Figures 2E–H**). In line with this finding, knockdown of FBXO43 reduced the expression of Ki-67, a biomarker of proliferation (**Figures 3E, F**). Taken together, knockdown of FBXO43 inhibits HCC cell proliferation.

### 3.3 Knockdown of FBXO43 inhibits the migration and EMT of HCC cells

To identify the role of FBXO43 in promoting the metastasis of HCC cells, the Transwell assay was performed. We found that silencing of FBXO43 significantly inhibited cell migration in Huh7 and LM3 cells (**Figures 3A, B**). Consistently, wound healing assay indicated that silencing of FBXO43 blocked the EMT of HCCs (**Figures 3C, D**). Furthermore, Western blot assays demonstrated that FBXO43 knockdown reduced the protein expression of N-cadherin and Vimentin and increased the expression of E-cadherin (**Figures 3E, F**). These results indicate that knockdown of FBXO43 inhibits the migration and EMT capacities of HCCs.

### 3.4 FBXO43 directly binds with CCND1 and promotes its stability

**Figure 4:**
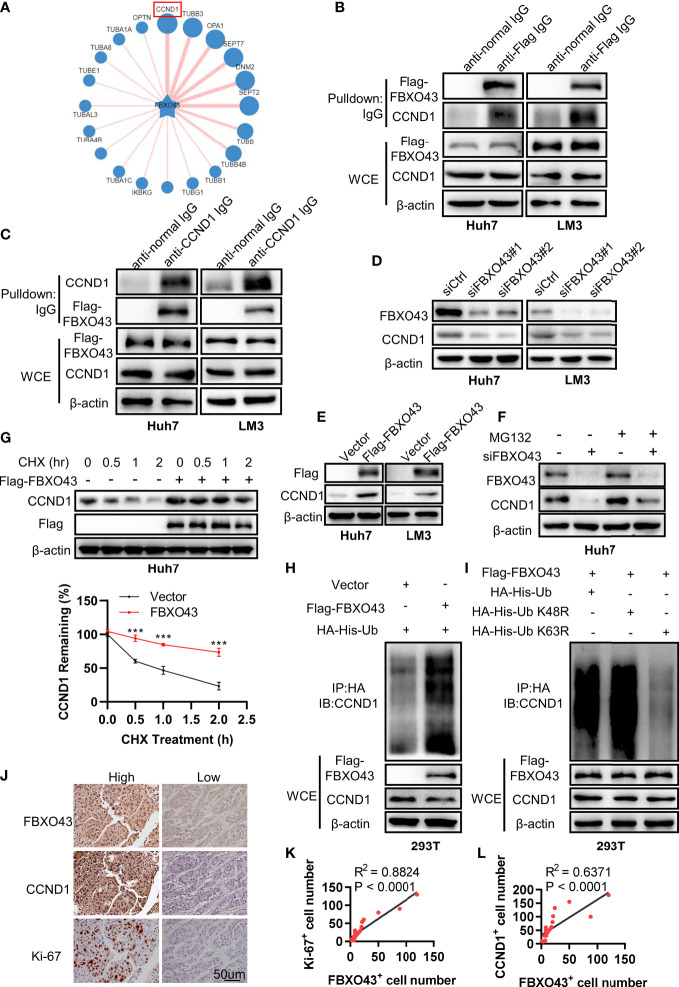
FBXO43 directly binds with CCND1 and promotes its stability. **(A)** Schematic diagram revealing the potential substrate of FBXO43 in the UbiBrower website. **(B)** Huh7 and LM3 cells were transfected with indicated plasmids for 48 hrs and then subjected to immunoprecipitation with anti-Flag or normal IgG antibody. The cell lysates were analyzed by Western blot. WCE, whole cell extract. **(C)** Huh7 and LM3 cells were transfected with indicated plasmids and then subjected to immunoprecipitation with anti-CCND1 or normal IgG antibody. The cell lysates were analyzed by Western blot. **(D)** The protein expression levels of FBXO43 and CCND1 were analyzed by Western blot in Huh7 and LM3 cells transfected with siCtrl or siFBXO43 for 48 hrs. **(E)** The protein expression levels of FBXO43 and CCND1 were measured by Western blot in Huh7 and LM3 cells transfected with indicated plasmids for 48 hrs. **(F)** Huh7 cells were transfected with siFBXO43 or siCtrl for 43 hrs and then treated with MG132 for 5 hrs before harvest for Western blot. **(G)** Huh7 cells were transfected with indicated plasmids for 48hrs and then treated with CHX (100μg/mL) for 0, 0.5, 1, and 2 hrs before harvest for Western blot. The quantification plot was based on scanning densitometry analysis using the Image J software. Relative protein levels were normalized to Vector group. **(H)** CCND1 was polyubiquitylated by FBXO43. HEK293T were transfected with indicated plasmids for 48 hrs and then subjected to immunoprecipitation with anti-HA antibody. The cell lysates were analyzed by Western blot. **(I)** FBXO43 mediated K63-linked polyubiquitination of CCND1. HEK293T cells were transfected with the FBXO43 plasmids along with WT-ubiquitin or its mutants (K63R and K48R) for 48 hrs and then subjected to immunoprecipitation with anti-HA antibody. The cell lysates were analyzed by Western blot. **(J)** Immunohistochemical staining was performed in HCC patients using anti-FBXO43, anti-CCND1, and anti-Ki-67 antibodies (n=20). FBXO43-positive, CCND1-positive, or Ki-67-positive cells were counted in 500 cells for each group. **(K, L)** Linear regressions were used to analyze the association between FBXO43- and CCND1-positive cells **(K)**, and FBXO43- and Ki-67-positive cells **(L)**. Two-tailed t-test was used. ***p<0.001.

To explore the potential mechanism of FBXO43 in promoting HCC growth, we predicted the potential substrates of FBXO43 through the UbiBrower website (http://ubibrowser.bio-it.cn/ubibrowser_v3/), among which CCND1 had the highest predicated score (**Figure 4A**). To evaluate whether FBXO43 binds with CCND1, Huh7 or LM3 cells were transfected with indicated plasmids and then analyzed by immunoprecipitation assay. We found that FBXO43 and CCND1 pulled down each other, demonstrating these two proteins were bound up with each other (**Figures 4B, C**). Furthermore, silencing of FBXO43 reduced CCND1 expression, whereas overexpression of FBXO43 upregulated CCND1 expression (**Figures 4D, E**). We next examined whether FBXO43 regulates degradation of CCND1 in the proteasome or by lysosomes. Chloroquine, a specific lysosome inhibitor, did not protect the CCND1 protein from degradation in Huh7 cells following FBXO43 knockdown, suggesting a lack of lysosome involvement (**Supplementary Figure 1**). However, MG132, a proteasome inhibitor, significantly blocked the FBXO43 knockdown-mediated the decrease of CCND1, suggesting that FBXO43 facilitates CCND1 stability (**Figure 4F**). Moreover, overexpression of FBXO43 dampened the degradation of CCND1 in Huh7 cells treated with CHX (**Figure 4G**). *Via in vivo* ubiquitination assay, overexpression of FBXO43 could increase the polyubiquitination of CCND1 (**Figure 4H**). To further characterize the type of ubiquitin chains involved in FBXO43-mediated CCND1 polyubiquitination, two ubiquitin mutants (K48R and K63R) were used along with the WT ubiquitin. The *in vivo* ubiquitination assay indicated that a K63R ubiquitin mutant significantly attenuated the formation of polyubiquitin chain to CCND1 compared with both WT and a K48R mutant form of ubiquitin (**Figure 4I**). Meanwhile, the protein expression levels of FBXO43, CCND1, and Ki-67 in 20 HCC patients were examined by IHC staining (**Figure 4J**). We found an apparent positive correlation between FBXO43 and CCND1 or Ki-67 in HCC (**Figures 4K, L**). Taken together, these findings indicate that FBXO43 binds and promotes CCND1 stability by polyubiquitination.

### 3.5 FBXO43-mediated cell proliferation and migration are dependent on CCND1 in HCC cells

**Figure 5:**
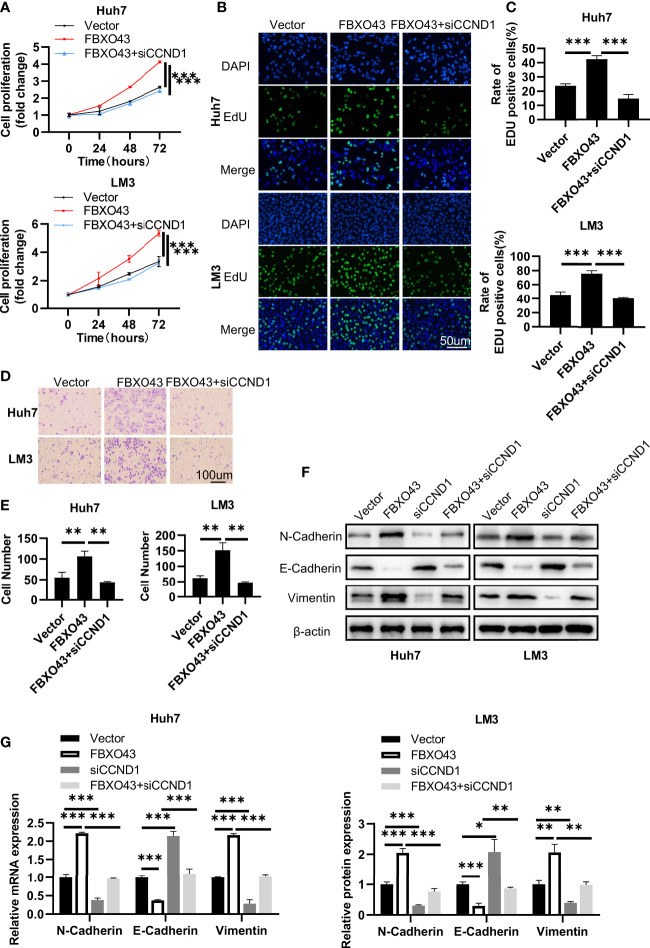
Knockdown of CCND1 blocks FBXO43-mediated cell proliferation and migration in HCC cells. **(A–C)** The cell proliferation levels were analyzed in Huh7 and LM3 cells transfected with Vector, Flag-FBXO43 or Flag-FBXO43+siCCND1 by MTT assay **(A)** and EdU incorporation assay **(B, C)**. **(D, E)** The cell migration levels were analyzed by Transwell assays in Huh7 and LM3 cells treated as in **(A)**. **(F, G)** Huh7 and LM3 cells were transfected with Vector, Flag-FBXO43, siCCND1 or Flag-FBXO43+siCCND1 for 48 hrs and then lysed. The protein and mRNA expression of EMT-associated markers was measured by Western blot **(F)** and qPCR **(G)**. One-way ANOVA with Tukey’s multiple comparisons test was used. *p<0.05, **p<0.01, ***p<0.001.

Then, rescue experiments were used to confirm that the oncogenic effects of FBXO43 were dependent on CCND1. We found that silencing of CCND1 could significantly impair the proliferation-promoting effect of FBXO43 overexpression using MTT and EdU incorporation assay (**Figures 5A–C**). Meanwhile, knockdown of CCND1 dramatically blocked FBXO43-mediated cell migration (**Figures 5D, E**). Furthermore, Western blot and qPCR assays demonstrated that the upregulation of mesenchymal markers including N-cadherin and Vimentin, and downregulation of epithelial marker E-cadherin induced by FBXO43 overexpression were also rescued by siCCND1 (**Figures 5F, G**). These results indicated that FBXO43-mediated cell proliferation, migration, and EMT are dependent on CCND1.

## 4 Discussion

Liver cancer, a very common malignant tumor disease, seriously endangers human health due to the poor treatment effects in the late stage, so it is urgent to find novel biomarkers for the early diagnosis of HCC. In recent years, the emergence of molecular targeted drugs such as sorafenib has provided a new direction for the treatment of liver cancer. Although many molecular targeted drugs have entered the stage of clinical trials, they have not received good feedback due to the complexity of cancers. Therefore, exploring the explicit mechanism of HCC is helpful for the research of molecular targeted drugs.

FBXO43, a member of F-box proteins involved in the occurrence and development of many cancers by regulating cell proliferation, apoptosis, invasion, and metastasis, has been initially demonstrated as an inhibitor of APC/C to ensure the normal progress of the meiotic division (6). Studies have reported that FBXO43 could promote tumor growth as a prognostic factor in breast cancer (16). A recent study suggested that FBXO43 promotes the progression of cholangiocarcinoma through the PI3K/Akt signaling axis (17). However, the intrinsic function and molecular mechanism of FBXO43 in HCC are still unknown. In this study, we firstly found that FBXO43 was highly expressed in HCC patients and was associated with poor clinicopathological features in the TCGA cohort. This finding was confirmed by HCC tissues from our center. In addition, HCC patients with high expression of FBXO43 had shorter OS and DFS. The multivariate Cox regression analysis showed that FBXO43 was an independent prognostic risk factor of HCC patients. These results revealed the potential diagnosis and prognostic value of FBXO43.

Cell proliferation and migration ability are important biomarkers in diagnosis and prognostic evaluation (18). Therefore, we observed that knockdown of FBXO43 inhibited HCC proliferation, migration, and EMT *in vitro*. To further elucidate the exact mechanism of the regulatory effect of FBXO43 on HCC, we predicted and verified that FBXO43 could directly interact with CCND1 by co-IP assays. CCND1, a cell cycle checkpoint in charge of G1-to-S transition, plays a crucial role in the regulation of cell proliferation and migration (19). Overexpression of CCND1 by post-translation is common in HCC. CCND1 ubiquitination is associated with E3 ubiquitin ligases including FBXO4 (20), FBXO31 (21), β-TRCP (22), and APC/C (23), while deubiquitination is associated with ubiquitin-specific protease (USP) family including USP2a (24), USP5 (25), USP10 (26), and USP22 (27). In the current study, we found that FBXO43 directly binds and promotes CCND1 stability by polyubiquitination. Future studies will be performed to explore how FBXO43 takes part in the ubiquitination modification of CCND1.

**Figure 6:**
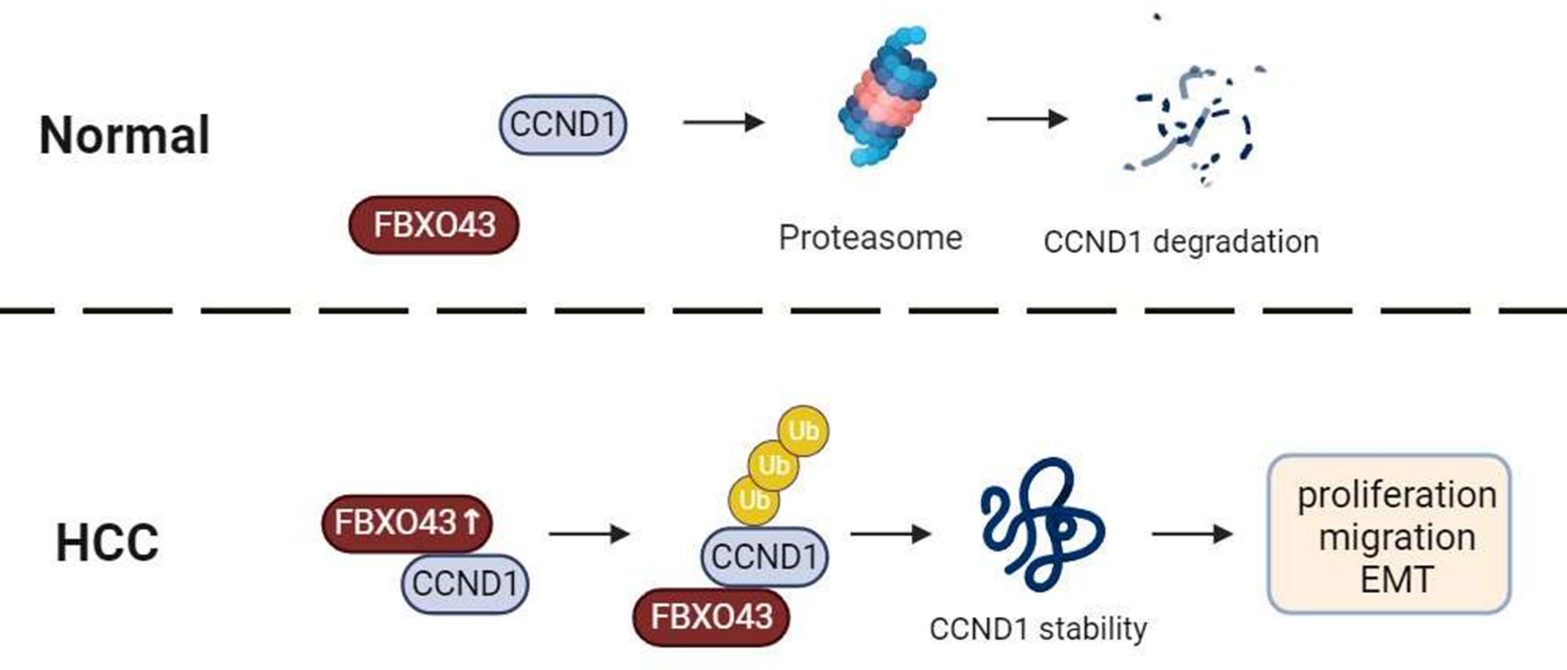
A graphical abstract of the role of FBXO43 in HCC. FBXO43 interacted with CCND1 and promoted its stability by polyubiquitination, leading to HCC cell proliferation, migration and EMT.

Overall, we firstly defined that FBXO43 acts as an onco-promoter and a biomarker of prognostic value in the HCC. FBXO43 interacted and promoted CCND1 stability by polyubiquitination, leading to HCC cell proliferation, migration and EMT (**Figure 6**). Future research will focus on the development of molecular targeted drugs for FBXO43 to prevent the progression, invasion, and metastasis of HCC.

## Data availability statement

The original contributions presented in the study are included in the article/**Supplementary Material**. Further inquiries can be directed to the corresponding authors.

## Ethics statement

The studies involving human participants were reviewed and approved by the Ethics Committee of the Southwest Hospital, Third Military Medical University. The patients/participants provided their written informed consent to participate in this study.

## Author contributions

X-JW, C-MX and J-PG designed the study. C-ML, JZ, WW, ZZ, FL, and DW performed the experiments. C-MX wrote the manuscript. All authors contributed to the article and approved the submitted version.
