# Supplementary material for: FBXO43 increases CCND1 stability to promote hepatocellular carcinoma cell proliferation and migration
Source: Front Oncol. 2023 Mar 3;13:1138348. doi: 10.3389/fonc.2023.1138348 (PMC10020529; doi:10.3389/fonc.2023.1138348)
Supplement: Supplementary file 1 [file Image_1.pdf]

Supplementary Material

FBXO43 increases CCND1 stability to promote hepatocellular carcinoma cell proliferation and migration

Chun-Ming Li[1], Jie Zhang[2], Wu Wu[1], Zhu Zhu[1], Feng Li[3], Di Wu[2], Xiao-Jun Wang[2*], Chuan-Ming Xie[2*], Jian-Ping Gong[1*]

[1]Department of Hepatobiliary Surgery, The Second Affiliated Hospital of Chongqing Medical University, Chongqing 400010, China

[2]Key Laboratory of Hepatobiliary and Pancreatic Surgery, Institute of Hepatobiliary Surgery, Southwest Hospital, Third Military Medical University (Army Medical University), Chongqing 400038, China

[3]Department of Hepatobiliary Surgery, The Third Affiliated Hospital of Chongqing Medical University, Chongqing 401120, China

*** Correspondence:**
Jian-Ping Gong, E-mail: gongjianping11@126.com

Chuan-Ming Xie, E-mail: cmxie@tmmu.edu.cn; chuanming506@126.com; Tel: +86 23 68765809, Fax: +86 23 68765809.

Xiao-Jun Wang, E-mail: yh830@163.com.

1 Supplementary Figures and Tables

1.1 Supplementary Fig. 1

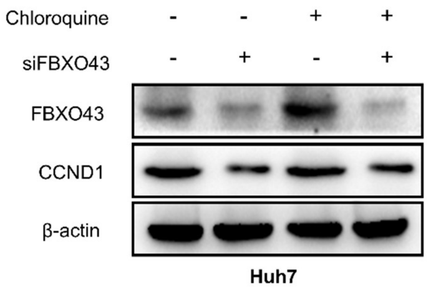

Supplementary Fig. 1. FBXO43 regulates CCND1 without the involvement of lysosomes

The Western blots to detect the level of CCND1 in siCtrl- or siFBXO43-treated Huh7 cells with or without a lysosome inhibitor (50 µM chloroquine).
